# Functional Characterization of *GhACX3* Gene Reveals Its Significant Role in Enhancing Drought and Salt Stress Tolerance in Cotton

**DOI:** 10.3389/fpls.2021.658755

**Published:** 2021-08-10

**Authors:** Margaret L. Shiraku, Richard Odongo Magwanga, Xiaoyan Cai, Joy Nyangasi Kirungu, Yanchao Xu, Teame Gereziher Mehari, Yuqing Hou, Yuhong Wang, Stephen Gaya Agong, Renhai Peng, Kunbo Wang, Zhongli Zhou, Fang Liu

**Affiliations:** ^1^Zhengzhou Research Base, State Key Laboratory of Cotton Biology, Institute of Cotton Research, Chinese Academy of Agricultural Sciences, Anyang, China; ^2^School of Biological and Physical Sciences (SBPS), Main Campus, Jaramogi Oginga Odinga University of Science and Technology (JOOUST), Bondo, Kenya; ^3^Anyang Institute of Technology, Anyang, China

**Keywords:** cotton, acyl-coenzyme A oxidase 3, peroxisomal, acetyl-CoA, overexpression, VIGS

## Abstract

The acyl-coenzyme A oxidase 3 (ACX3) gene involved in the β-oxidation pathway plays a critical role in plant growth and development as well as stress response. Earlier on, studies focused primarily on the role of β-oxidation limited to fatty acid breakdown. However, ACX3 peroxisomal β-oxidation pathways result in a downstream cascade of events that act as a transduction of biochemical and physiological responses to stress. A role that is yet to be studied extensively. In this study, we identified 20, 18, 22, 23, 20, 11, and 9 proteins in *Gossypium hirsutum*, *G. barbadense*, *G. tomentosum*, *G. mustelinum*, *G. darwinii*, *G. arboretum*, and *G. raimondii* genomes, respectively. The tetraploid cotton genome had protein ranging between 18 and 22, while diploids had between 9 and 11. After analyzing the gene family evolution or selection pressure, we found that this gene family undergoes purely segmental duplication both in diploids and tetraploids. W-Box (WRKY-binding site), ABRE, CAAT–Box, TATA-box, MYB, MBS, LTR, TGACG, and CGTCA-motif are abiotic stress *cis-*regulatory elements identified in this gene family. All these are the binding sites for abiotic stress transcription factors, indicating that this gene is essential. Genes found in G. *hirsutum* showed a clear response to drought and salinity stress, with higher expression under drought and salt stress, particularly in the leaf and root, according to expression analysis. We selected *Gh_DO1GO186*, one of the highly expressed genes, for functional characterization. We functionally characterized the *GhACX3* gene through overexpression and virus-induced gene silencing (VIGS). Overexpression of this gene enhanced tolerance under stress, which was exhibited by the germination assay. The overexpressed seed growth rate was faster relative to control under drought and salt stress conditions. The survival rate was also higher in overexpressed plants relative to control plants under stress. In contrast, the silencing of the *GhACX3* gene in cotton plants resulted in plants showing the stress susceptibility phenotype and reduced root length compared to control. Biochemical analysis also demonstrated that *GhACX3*-silenced plants experienced oxidative stress while the overexpressed plants did not. This study has revealed the importance of the ACX3 family during stress tolerance and can breed stress-resilient cultivar.

## Introduction

Drought and salt stresses are environmental factors that affect plant growth and development (Su et al., 2020), reproduction, ultimately, and reduce crop yield. The deleterious effect on crop yield is magnified with the ever-changing climatic situation (Allen et al., 2010). Plants have developed elaborate mechanisms that initiate physiological, morphological, and molecular changes to adapt to stress conditions (Ahuja et al., 2010). Stress-responsive genes are known to regulate these mechanisms. Notably, the *ACX3* gene is involved in central metabolisms that respond to stress.

The acyl coenzyme A oxidase (*ACX*) gene family is a diverse family of enzymes involved in the β- oxidation pathway of fatty acids (Graham and Eastmond, 2002). These genes are present in all organisms, plants (Grossi et al., 1995; De Bellis et al., 2002; Graham and Eastmond, 2002; Li, 2005), yeast (Kim and Kim, 2018), nematodes (Joo et al., 2010), microbial organisms (Reiser et al., 2010), and mammals (Eaton et al., 2015). Acyl CoA oxidases (*ACX*s) participate in the initiation step of the β-oxidation pathway; thus, they are essential genes (Kindl, 1993). Acyl coenzyme A oxidase (*ACX*) genes, in plants, are located in the peroxisome, whereas in animals, in the mitochondria (Reddy and Hashimoto, 2002). *ACXs* genes differ from one another, some are homodimers, and others occur in a single copy. The family also differs according to catalytic positions and the precision of the substrate they catabolize. For instance, in Arabidopsis, there are six isoforms of ACX, but each act on different (sized) carbons present in the acyl-CoA substrate. *AtACX1* catalyzes the breakdown of medium and long-chain acyl-CoA, *AtACX2* prefers long chain acyl-CoA, and *AtACX3* prefers medium-chain acyl-CoA while AtACX4 acts on short-chain acyl-CoA. Experimentally, *AtACX5* and *AtACX6* have not been characterized (Kim and Kim, 2018). *ACXs* have a significant role not limited to primary and secondary metabolism, growth, and development but also in responses to abiotic and biotic stresses (Hu et al., 2012). β-oxidation results in phytohormone production like jasmonic acid, indole acetic acid, and salicylic acid involved in stress responses (Kim et al., 2009; Sandalio, 2015). In addition, hydrogen peroxide produced in this process serves as a stress signal molecule (Kindl, 1993). Another scientist found that genes encoding *ACX*s are distributed in most plant tissues, including leaves, roots, inflorescence, and bolts; this is because β-oxidation of fatty acid occurs throughout plants’ life cycles (Eastmond et al., 2000). It has been demonstrated that, through fatty acid β-oxidation, the plant utilizes its storage reserves and initiates stress signaling (Li, 2005). Acetyl-CoA produced in β-oxidation is important in histone acetylation. These were clearly demonstrated by an acx4-4 mutant immunoblotting assay conducted to identify the effect of the acx4-4 mutant on histone acetylation and found to impair histone acetylation relative to WT. The above findings indicate that gene mutations in β-oxidation reduce acetyl-CoA in peroxisomes, which can lower cytosol acetyl-CoA levels and reduce histone acetylation and increase DNA methylation (Wang et al., 2019). Acetyl-CoA produced during β-oxidation drives a cycle of tricarboxylic acid (TCA) for the aerobic production of ATP (de la Peña Mattozzi et al., 2010). Therefore, *ACXs* genes are critical in the production of energy under aerobic conditions, gene epigenetic modification, and transcription of stress-responsive genes.

The role of ACX genes as a protein that impedes stress has been examined in other plants like cucumber (Apoprotein et al., 1986), *Arabidopsis thaliana* (Kim and Kim, 2018), pumpkins (De Bellis et al., 2002), tomato (Li, 2005), maize (Hooks et al., 1996), and barley (Grossi et al., 1995), among other plants, to understand *ACX* genes involvement during the abiotic and biotic stress response. For instance, in maize, it has been demonstrated that, during glucose starvation, which can occur due to drought, ACXs enzyme activity increased, subsequently upregulating β-oxidation activity as well (Hooks et al., 1996). In Arabidopsis, during abiotic stress, *AtACX3* gene mechanisms increased and resulted in a high concentration of hydrogen peroxide (Froman et al., 2002). The *ACX3* gene is upregulated during foliar senescence in response to stress in plants like barley (Grossi et al., 1995) and cucumber (Kindl, 1993). Another study demonstrated that these genes participate in the oxidation of toxic short-chain fatty acids produced during abiotic stress like drought (Hayashi et al., 1999). In a study to identify the effect of overexpressing *GaJAZ1* in upland cotton, *ACX3* (Gh_D01G0186) was highly upregulated. Overexpression of the *G. arboreum JAZ1* enhanced salt stress tolerance in cotton (Ali and Baek, 2020).

Cotton (*G. hirsutum*) is predominantly, on a commercial scale, the main source of fiber worldwide. However, due to drastic climate change, drought and salt stress have negatively affected cotton production resulting in reduced yield and quality fiber. Previous researchers have demonstrated that plants can sense and respond to abiotic stresses. Different aspects, including modifying protein and enzyme production, diversely control plant response to abiotic stress. ACX3 is an enzyme but plays a role in stress response. Therefore, understanding *ACX3* nuclear modification and its role in response to abiotic stress tolerance is essential. To get some insight into the function of *GhACX3* in improving drought and salt tolerance in cotton, we undertook the functional characterization of this gene through overexpression and knockdown of this gene in Arabidopsis and cotton, respectively. We analyzed the effect of salt and drought stress in overexpressed and knockdown plants. We undertook bioinformatics analysis to understand the structure of the gene and motif and *cis-*regulatory elements present. We also studied the evolutionary relationship of this gene in wild and domesticated cotton species and other plant species. Our study revealed this gene as a candidate for cotton genetic modification and provided insights into cotton’s stress tolerance mechanisms.

## Materials and Methods

### Identification of ACX3 Protein and Sequences Retrieval

We obtained the ACX3 protein sequences of different plant species from published genome databases utilizing the BlastP program, with the AtACX3 protein sequence obtained from Arabidopsis as the query. The ACX3 proteins were obtained for the plant species; *Arabidopsis thaliana* (Cao et al., 2011), *Theobroma cacao* (Argout et al., 2011), *Populus trichocarpa* (Tuskan et al., 2006), *Gossypium hirsutum* (Wang L. et al., 2019), *Gossypium barbadense* (Wang L. et al., 2019), *Gossypium tomentosum* [27], *Gossypium mustelinum, Gossypium darwinii* (Chen C. et al., 2020), *Gossypium arboreum* (Wang et al., 2019), and *Gossypium raimondii* (Wang H. et al., 2012; Wang K. et al., 2012). This was to allow for the analysis of the phylogeny of the proteins encoded by the *ACX3* genes. The Pfam database was then employed to query all the genes, the genes with the ACX3-conserved domain PF01756 were then selected. In addition, the physiochemical properties were analyzed by using an online tool ExPASy Server^1^ (Swiss Institute of Bioinformatics).

### Phylogenetic Tree, Collinearity, and Selection Pressure Analysis

In order to elucidate the phylogeny of the proteins encoded by the *ACX3* genes, all the identified proteins from the various plants were aligned by using a free online tool, the ClustalX package. The phylogenetic tree was then constructed using MEGA 7 (Horiike, 2016), where the evolutionary history of the different proteins was determined using the neighbor-joining method (Saitou and Nei, 1987). Furthermore, in the analysis of the collinearity of the *ACX3* genes from the three cotton genomes, the gff3 files containing the ortholog and paralog genes of *G. hirsutum, G. arboretum*, and *G. raimondii* were downloaded from the cotton genome database^2^. These files were then submitted to TbTools (Chen C. et al., 2020) to draw the gene family’s collinearity map. The non-synonymous (Ka)/synonymous (Ks) mutation ratio (Ka/Ks), which infers the selection pressure of proteins in a particular gene family, the CDS sequences, gene IDs, and homologous genes of *G. hirsutum, G. arboreum*, and *G. raimondii* were employed to analyze the type of selection pressure within the cotton ACX3 proteins.

### Chromosome Allocation, Gene Structures, Conserved Motif, GO Analysis, and *Cis-*Acting Regulatory Element Identification

The position- and the chromosome-specific genes were then mapped using TbTools (Chen C. et al., 2020). The nucleotide sequence 2000 bp upstream of the transcription site of the ACX3 of *G. hirsutum, G. arboreum*, and *G. raimondii* were submitted to the PlantCARE database to ascertain the associated *cis-*regulatory elements (Lescot, 2002). To predict the structure of these genes and the position of the introns and exons, an online tool Gene Structure Display Server (GSDSv2)^3^ (Hu et al., 2015) was used. Moreover, the preserved motifs were identified by the online tool MEME Suite^4^ (Bailey et al., 2009; Kirungu et al., 2020). Finally, gene ontology helps in understanding the possible pathways in which the proteins encoded by the *ACX3* genes are involved. We employed the GO analysis toolkit and database, AgriGO v2.0, to conduct gene ontology enrichment analysis to identify the gene family’s molecular function, cellular component, and biological function (www.bioinfo.cau.edu.cn/agriGO) (Tian et al., 2017).

### Plant Material and Treatment

The seeds of *G. hirsutum*, Marie-Galante 85, a semi-wide variety with higher tolerance to various abiotic stress factors (Kirungu et al., 2019; Nyangasi et al., 2019; Xu et al., 2019) was used. The delinting was done using sulfuric acid and afterward grown on absorbent paper for 48 h. After germination, the seedlings were then transferred to a Hoagland nutrient-rich solution (Hoagland and Arnon, 1950) in the greenhouse, with conditions set at 16 h light/8 h dark, the temperature at 28°C in the day and 25°C at night. A drought and salt stress simulation was done at the three-leaf stage by adding 300 mM of sodium chloride for salt treatment and 17% (w/v) PEG-6000 for drought treatment to the Hoagland solution (Lu et al., 2018; Magwanga et al., 2019c; Yang et al., 2019a; Magwanga and Kirungu, 2020). Thereafter samples were taken from the leaf, stem, and root tissues at 0, 12, 24, and 48 h post stress exposure, as previously explained by Magwanga et al. (2019a). To further understand the possible roles of the *ACX3* genes, one of the highly upregulated genes was then transformed into the model plant: Arabidopsis ecotype Col-0 plants. The Arabidopsis seeds used in this study were surface sterilized and grown on 50% MS medium, and grown in the greenhouse with conditions set as explained by Yang et al. (2019b).

### Isolation of RNA and qRT-PCR Analysis

Total RNA was isolated from plant tissues using an RNAprep Pure Plant kit (Tiangen, Beijing, China) as per the manufacturer’s instructions. We further determined the RNA quality and concentration by use of agarose gel electrophoresis and spectrometric analysis (Kirungu et al., 2018; Lu et al., 2018, 2019). The RNA was then used for cDNA synthesis using a cDNA conversion kit obtained from Transgene, Beijing, China (EasyScript First-strand cDNA Synthesis SuperMix). The applied biosystems 7500 real-time PCR system performed the RT-qPCR (Zhang et al., 2020), using 2 × AceQ^TM^ Universal SYBR qPCR Master Mix (Vazyme Biotech, Nanjing, China). The samples for analysis were used in three independent biological and technical replicates. All the gene-specific primers are shown in the Supplementary Materials. The transcript variation was calculated using the 2^–ΔΔC^*^T^* method (Livak and Schmittgen, 2001).

### Subcellular Localization of GFP–Tagged ACX3 Protein

To determine the subcellular localization of the ACX3 protein, we used the online tool WoLF PSORT (Hortona et al., 2006) and the TargetP1.1 server (Emanuelsson et al., 2000). In order to confirm the subcellular localization, a GhACX3-coding DNA sequence was amplified using its specific primer; 5′-GAGAACACGGGGGACTCTAGAATGGAACAAGCTTTCAA AAGA-3′ and 5′-ACCCATGTTA ATTAAGGATCCA ACCGAAGACCAAGCGTTTGC-3′. The PCR product was infused and ligated into a p2300-eGFP vector under the regulation of the CaMV 35S promoter. After that, the product was transformed into *Agrobacterium tumefaciens* strain GV3101, and empty vector p2300-eGFP was used as control (CK). The agrobacterium suspensions were infiltrated into the abaxial leaf side of 4-week-old *N. benthamiana* plants. Afterward, they were put in the dark for 48 h, then the GFP signal was observed under a fluorescence confocal microscope (Leica DMi8 Microscope).

### Plasmid Construction of Overexpressed GhACX3 and Stress Treatment

The open reading frame of GhACX3 2013 bp was amplified using *G. hirsutum* cDNA, 5′GAGAACACGGGGGACTCTAGAATGGAACAAGCTTTCA AAAGAACCCAAATT 3′ and 5′GGACTGACCACCC GGGGATCCTTAAACCGAAGACCAAGCGTTTGCTTCAAT3′ primers through PCR. It was then inserted in the Xbal1 and BamH1 sites of the PBI121 vector and transformed to *Agrobacterium tumefaciens* strain GV3101. The *A. thaliana*, ecotype Colombia-0 (Col-0), was transformed by adopting the floral dip method (Hu et al., 2019). Transformed T1 and T2 generations were selected on 50% MS medium with kanamycin. The T3 homozygous generations were then selected from the T2 generation through semi-quantitative RT-PCR and qRT-PCR analysis. Salt and drought treatment was done on the T3 generation. Drought stress was simulated by withholding water for 8 and 12 days, while salt stress was initiated by watering the plants with 250 Mm of sodium chloride (NaCl) for 8 and 12 days (Magwanga et al., 2018a). The plants that were relieved of drought stress by being re-watered to determine the survival rate.

### Root Stress Tolerance Assay

The T3 generation lines and WT were used for the root assay. Sterilized seeds of WT, L1, L2, and L3, the T3 generation lines chosen through semi-quantitative PCR and RT-qPCR, were grown in plates with 50% MS medium augmented with different concentrations of NaCl and mannitol. The concentration for salt stress was NaCl (0, 75, and 150 mM) and for drought stress, mannitol (0, 100, and 300 mM). After 10 days, the germination rate was determined. To evaluate root length, L1, L2, L3, and WT Arabidopsis seeds were grown in 50% MS media for 6 days then transferred to 50% MS medium with varying concentrations of mannitol (0, 100, and 300 mM) and NaCl (0, 75, and 150 mM) (Ma et al., 2019). The root length was then measured after 7 days of stress exposure; photographs were taken alongside the measurements.

### Virus-Induced Gene Silencing (VIGS) and Stress Treatment

Fragment of the coding DNA sequence of *GhACX3* 2013 bp was used to design the specific primer, forward sequence: GTGAGTAAGGTTACCGAATTCCCAATCATCTGCTCCAAT CC and reverse sequence: CGTGAGCTCGGTACCGGATCCGA ACACCTTCCCTCCCCTAA and amplified by PCR. The product was then cloned into the EcoR1 and BamH1 sites of the plasmid tobacco rattle virus vector (pTRV) to generate pTRV: ACX3. The *A. tumefaciens* LBA4404 strain was transformed using the freeze and thaw method (Jyothishwaran et al., 2007). The preparation of the bacteria inoculum and inoculation to the cotton plant’s cotyledon was done as described by Corbin et al. (Yang et al., 2019b). At the three-leaf stage after inoculation, drought and salt stress was initiated. Drought and salt stress concentrations were 17% (w/v) PEG-6000 and 250 mM of NaCl, respectively. pTRV: 00 (empty vector) and wild-type (untreated) plants were used as a negative control (Yang et al., 2019b). A phytoene desaturase (PDS) was used as a phenotypic marker to ascertain the effectiveness of the knockdown of the gene. We further conducted RT-qPCR to determine silencing efficiency.

### Assessment of the Physiological and Morphological Plant Features Under the Conditions of Drought and Salt Stress

All samples were collected with three biological replicates before treatment, and at 48 h post stress treatment. We analyzed the physiological and morphological traits of the plants. The physiological characteristics analyzed were excised leaf water loss (ELWL), relative leaf water content (RLWC), and cell membrane stability (CMS) which were measured through ion leakage as described by previous researchers (Clarke, 1986; Pietragalla and Mullan, 2012; and Ilík et al., 2018). At the same time, the morphological traits encompassed plant height (PH), root length (RL), shoot fresh weight, and root fresh weight were evaluated as previously outlined (Kirungu et al., 2020).

### Biochemical Analysis, Chlorophyll Content Determination, and Diaminobenzidine (DAB) Staining

By quantifying the oxidant and antioxidant levels in control, silenced, and transformed plants, we were able to examine the effect of simulated drought and salt stress on oxidant and antioxidant activities. The oxidants activities assayed were malondialdehyde (MDA) and hydrogen peroxide (H_2_O_2_), while the antioxidants were peroxidase (POD) and catalase (CAT). In all the assays, 0.1 g of ground tissue was used in three biological replicates. Extraction and spectrometric analysis of the oxidants and antioxidants was carried out using their respective assay kits supplied by Beijing Solarbio Science & Technology, China. In determining chlorophyll content, 0.1 g of Arabidopsis rosette leaves were immersed in 1.5 mL of 95% ethanol and put in a dark environment for 48 h at room temperature. The absorbance of the chlorophyll extracted was measured at 649 and 665 nm (Lichtenthaler and Buschmann, 2001). Whereas DAB (3,3′-Diaminobenzidine) staining was done using a DAB staining kit (Solarbio LIFE SCIENCE) with reference to the manufacturer’s instructions. Thereafter, images of the stained leaves were taken.

### Expression Profiling of Stress-Responsive Genes

We evaluated the expression level of the abiotic stress-responsive genes *GhMYB*, *GhG-T2*, *GhP5CS*, *AtAB15*, *AtP5CS*, and *AtRD22* through RT-qPCR in the tissues of control, VIGS, and overexpressed plants under drought and salt stress conditions, using *AtActin2* and *GhActin7* as the endogenous control. As demonstrated by previous researchers, these genes are highly upregulated in various cotton and Arabidopsis plants’ tissues and enhance drought and salt stress tolerance.

### Statistical Analysis

The experiments were done in three biological replicates, and the data were statistically analyzed by the analysis of variance (ANOVA) procedure (Molugaram and Rao, 2017), using GraphPad Prism. The least significant difference test (*P* ≤ *0.05*) was used for mean comparison.

## Results

### Identification of ACX3 Proteins

The ACX3 domain PF01756, obtained from the Pfam database, was used to identify the proteins in the cotton sub-genomes. In *G. hirsutum* (AD) _1_, we identified 20 proteins, in *G. barbadense* (AD) _2_, 18 proteins, in *G. tomentosum* (AD) _3_, 22 proteins, *G. mustelinum* (AD) _4_, 23 proteins, *G. darwinii* (AD) _5_, 20 proteins, in *G. arboreum* A_2_, 11 proteins, and *G. raimondii* D_5_, nine (9) proteins. The tetraploid proteins (AD) _1_–(AD) _5_ outnumbered diploid A_2_ and D_5_ due to gene replication over the years. The molecular weight in *G. hirsutum* proteins ranged between 17 and 78.075 kDa, while in *G. raimondii* and *G. arboreum* it ranged between 18.054 and 77.329 kDa. The isoelectric point ranged from 4.972 to 8.672, the CDS GC percentage content was an average 44.5%, and the grand average of the hydropath, Gravy, was negative in all the proteins (Supplementary Table 1).

### Evolutionary Analysis, Gene Duplication, and Selection Pressure of the ACX3 Protein

**FIGURE 1:**
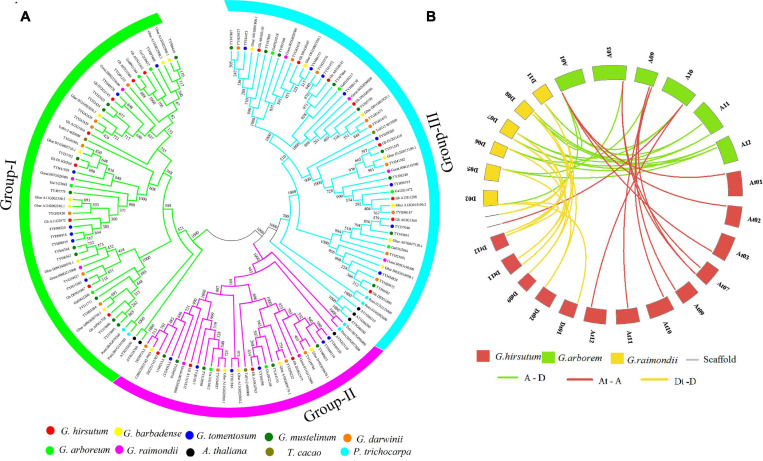
**(A)** Phylogenetic tree analysis of the tetraploid cotton, diploid cotton, and other plant proteins encoded by the ACX3 proteins; *Arabidopsis thaliana, Theobroma cacao, Populus trichocarpa, Gossypium hirsutum, Gossypium tomentosum, Gossypium barbadense, Gossypium mustelinum, Gossypium darwinii, Gossypium raimondii*, and *Gossypium arboreum*. The different colors indicate the different ACX3 proteins. The phylogenetic tree was generated from an alignment of the ACX3 proteins using the neighbor-joining method in the MEGA 7 software package. **(B)** Collinearity analysis in *Gossypium hirsutum*, *Gossypium raimondii*, and *Gossypium arboreum*. Syntenic relationships among ACX3 genes from *G. hirsutum*, *G. raimondii*, and *G. arboreum*. Their chromosomes are indicated in different colors. The putative orthologous ACX3 genes between G. raimondii and G. arboreum are represented in green and between *G. raimondii* and *G. hirsutum* is represented by yellow and between *G. hirsutum* and *G. arboreum* is represented by red.

To determine the evolutionary relationship of the ACX3 protein, protein sequences of *Arabidopsis thaliana, Theobroma cacao, Populus trichocarpa, G. hirsutum, G. tomentosum*, *G. barbadense, G. mustelinum, G. darwinii, G. raimondii, and G. arboreum* were aligned. We constructed the phylogenetic tree, which showed the evolution relation of ACX3 proteins in the tetraploid cotton, diploid cotton, and the other plant species. The ACX3 proteins were clustered in three groups (clades) (Figure 1A). The *G. hirsutum* proteins were closely clustered to *G. darwinii* compared to other species. In contrast, *G. barbadense* and *G. mustelinum* proteins were closely clustered together; all these indicate a closer evolution relationship.

The principal causes of gene-family enlargement in cotton species are tandem and segmental replication, which occurs during polyploidization. Two or more genes, one after the other, on the same chromosome confirm tandem duplication events, while gene duplication is characterized as a segmental duplication event on multiple chromosomes. During evolution, duplicated genes undergo selection. The Ka/Ks ratio denotes the orientation and magnitude of the natural selection of protein-coding genes. A ratio greater than 1 means positive or Darwinian selection; less than 1 means selection purification or stabilization (acting against change); and a ratio of exactly 1 means selection neutral (i.e., no selection) (Kondrashov et al., 2002). In this study, we determined that this gene family undergoes purifying selection (Figure 1B and Supplementary Table 2).

### ACX Gene Structures, Domain, and Conserved Motif

**FIGURE 2:**
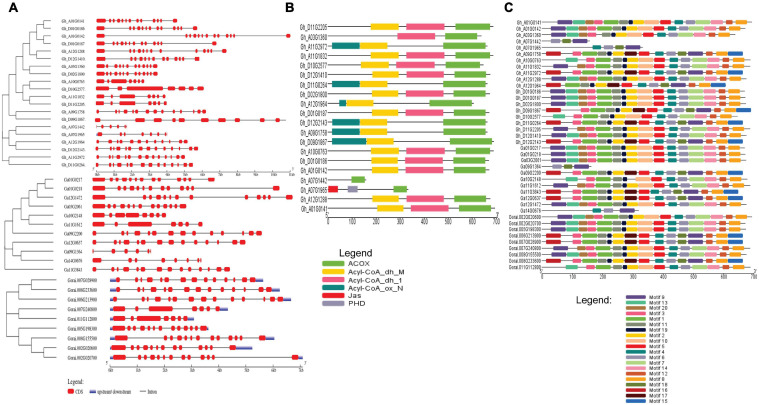
**(A)** Gene structure of cotton, *G. hirsutum, G. arborem*, and *G. raimondii*. **(B)** ACX3-conserved domains present in the *G. hirsutum* genes. **(C)** Motif for the *G. hirsutum, G. arboretum*, and *G. raimondii ACX3* genes. The identified motifs are denoted by different colors.

The gene structure of *G. hirsutum*, *G. arboreum*, and *G. raimondii* were analyzed to determine the exon and intron arrangement and CDS. The exons number ranged between 5 and 15 introns in a gene. In the analysis of the conserved motif, most of the genes had the same type of motif. For example, motifs 1, 2, and 8 were present in most genes indicating close evolution (Figures 2A–C).

### *Cis-*Acting Regulatory Element, Gene Ontology (GO), and Chromosome Mapping

**FIGURE 3:**
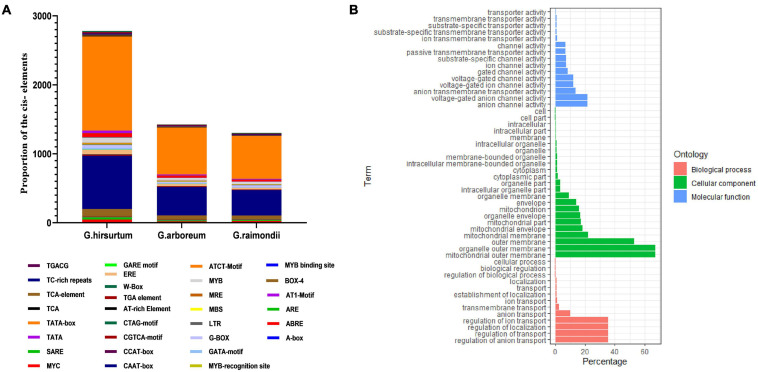
**(A)***Cis*-regulatory elements and Gene Ontology annotation. **(A)** Average number of *cis*-promoter elements in the regions of *G. hirsutum, G. raimondii*, and *G. arboreum ACX3* genes. The *cis*-promoters were analyzed in the 2 kb up/down stream promoter regions of the translation start site, using the PLACE database. **(B)** Gene Ontology (GO) annotation results for upland cotton *ACX3* genes. Various GO terms were predicted that indicated *ACX3* genes’ involvement in various biological processes, cellular component, and molecular functions.

Several ACX3 *cis-*regulatory elements associated with abiotic stress responsiveness were identified in *G. hirsutum* (AD_1_), *G. arboreum* (A_2_), and *G. raimondii* (D_5_). The identified *cis-*regulatory elements were W-Box (WRKY-binding site), ABRE, CAAT–Box, TATA-box, MYB, MBS, TGA, LTR, TGACG, CGTCA-motif, G-Box, and Box-4, among others (Figure 3A). These *cis-*regulatory elements were involved in phytohormones (abscisic acid, auxin hormone, and Meja) low-temperature responsiveness, light responsiveness, and a binding site for abiotic stress transcription factors. In order to predict the putative role of the proteins encoded by the *ACX3* genes in cotton, gene ontology provided basic information. In the analysis of gene ontology, all the three basic classifications as per gene ontology, cellular component (CC), biological processes (BP), and molecular functions (MF) were observed. The GO terms associated with molecular function were: GO: 0003997-Acyl-CoA oxidase activity, GO: 0016622-oxidoreductase activity, GO: 0003995-acyl-CoA dehydrogenase activity, and GO: 0050660-flavin adenine dinucleotide binding activities. Biological process GO terms were: GO: 0006632-fatty acid beta-oxidation, GO: 0055114-oxidation-reduction process, GO: 0006631-fatty acid metabolic process, and GO: 0008152-metabolic process. The cellular component was the peroxisome-GO: 00057777 (Figure 3B).

**FIGURE 4:**
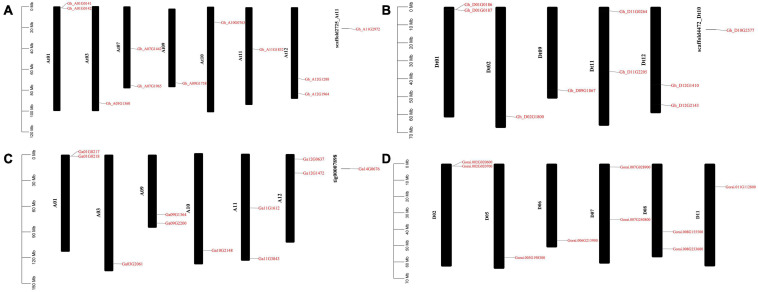
Chromosome mapping of ACX3 genes. **(A,B)** Chromosome mapping for the tetraploid cotton, *G. hirsutum*, **(C)** chromosome mapping for *G. arboretum*, a diploid cotton of the A genome. **(D)** Chromosome mapping for the diploid cotton, *G. raimondii* of the D genome. The genes are distributed on different chromosomes and chromosome number indicated.

GFF3 files containing chromosome position information of all the *ACX3* genes *in G. hirsutum, G. arboreum*, and *G. raimondii* were retrieved from the cotton functional genomic database, and TbTools was used for mapping. In *G. hirsutum*, the *ACX3* genes identified were mapped to seven chromosomes in A_t_ subgenomes, five chromosomes from Dt subgenomes, and two scaffolds. While *G. arboreum* and *G. raimondii* were mapped to chromosomes 1, 2, 3, 5, 6, 7, 8, 9, 10, 11, and 12 and one was localized in the scaffold (Figures 4A–D). We conducted GO enrichment analysis and identified the molecular function, biological process, and cellular component of these genes’ activities.

### Profiling of GhACX3 Gene Under Drought and Salt Stress

**FIGURE 5:**
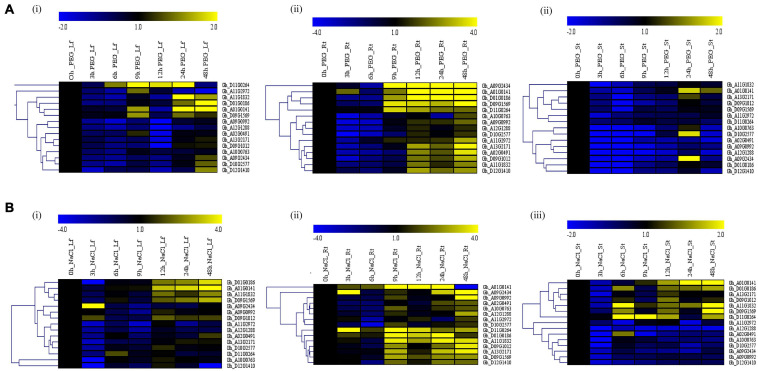
RT-qPCR analysis of the cotton *GhACX3* genes under drought and salt stress conditions. **(A)** Heatmap of the *GhACX3* genes expression under drought stress in the leaf (Lf), root (Rt), and stem (St). **(B)** Heatmap of the *GhACX3* genes expression under salt stress conditions in the leaf (Lf), root (Rt), and stem (St). Yellow represents a high expression of the genes, and blue represents a low expression of genes. Black represents no expression of the genes at a particular time.

We treated the *G. hirsutum* seedlings grown in the hydroponic solution for salt and drought stress by supplementing hydroponic water with 250 Mm of NaCl and 17% PEG-6000, respectively. Samples were collected at an interval of 3 h, starting from 0 to 12 h, then at 24 and 48 h from the leaf, stem, and root. RNA extracted from the tissues was converted to cDNA and used for RT-qPCR analysis. The expression of the *GhACX3* genes was differential (Figure 5). Under drought stress, most of the genes were highly expressed in the leaf and root. While under salt stress, most of the genes were highly upregulated in the leaf, stem, and roots. Even though the genes were upregulated differentially in all tissues, the roots’ expression was higher under both salt and drought stress. This was more evident when we evaluated the cloned *Gh_DO1GO186* gene expression in leaf, stem, and root organs; this gene was highly upregulated in the roots.

### Subcellular Localization and Knockdown of GhACX3 Reduced Cotton Plant Tolerance to Drought and Salt Stress

**FIGURE 6:**
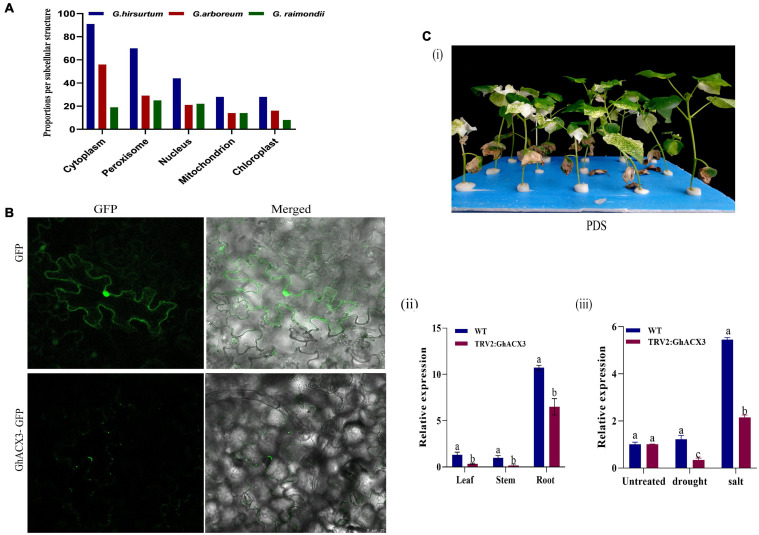
Subcellular localization and confirmation of silencing. **(A)** Subcellular localization in *G. hirsutum, G. arboretum*, and *G. raimondii* as predicted by WoLF PSORT. **(B)** Subcellular localization of GhACX3. GhACX3-GFP represents the gene and GFP represents the control that is an empty vector. **(C)** Confirmation of silencing. **(i)** Representative images of PDS **(ii)** expression of the silenced gene in the leaf, stem, and root. **(iii)** Relative expression of the *GhACX3* gene in normal condition (WT), empty vector (TRV2: 00), and silenced GhACX3 plants.

The online tool WoLF PSORT and the TargetP1.1 server predicted that this gene is located in the nucleus, mitochondrion, chloroplast, cytoplasm, and peroxisome (Figure 6A). To confirm the subcellular localization, *GhACX3* was transformed in Agrobacterium and inoculated in *N. benthamiana* leaves. It revealed that the proteins encoded by the *ACX3* genes were located within the cytoplasm (Figure 6B). Adaptation to stress requires cellular ion homeostasis involving net intracellular vacuolar compartmentalization without toxic ion accumulation in the cytosol. The balancing of the ions at the cytoplasm is critical in maintaining various cellular activities, and perhaps the ACX3 proteins could be playing a critical role. Moreover, in order to further elucidate the possible role of the ACX3 protein, the key gene was determined and knocked down in cotton. Furthermore, virus-induced gene silencing is an innovative tool that has been used to characterize genes (Kant and Dasgupta, 2019) functionally. In this study, we silenced the *Gh_DO1GO186* gene in cotton plants. Phytoene desaturase (PDS) was used as a phenotypic marker. Plants were inoculated with TRV2: PDS after 14 days, the TRV2: PDS-inoculated plant leaves showed a bleached phenotype (Figure 6Ci). This result is an affirmative indication that the knockdown vector was effective, and the *Gh_DO1GO186* gene knockdown was successful. We further conducted RT-qPCR to validate the knockdown efficiency; the expression level of *Gh_DO1GO186* in silenced plants was relatively lower than WT in leaf, stem, and root tissues, however, the knocked-down gene expression was significantly higher in the root compared to the leaf and or stem (Figure 6Cii); similarly, when the VIGS plants and wild-type were subjected to both drought and salinity stress, the induction level of the silenced gene was significantly reduced in the VIGS plants compared to the wild-type, and the expression pattern depicted that of non-treated plants, with the roots exhibiting significantly higher expression levels (Figure 6Ciii).

### Morphological and Physiological Evaluation of the VIGS Plants and Wild-Type

**FIGURE 7:**
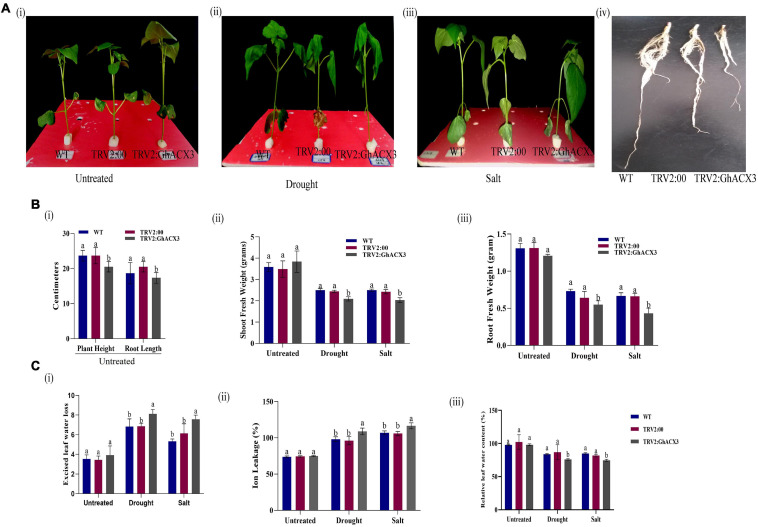
Phenotypic observation of the plants’ morphological and physiological evaluation. **(A) (i–iii)** Representative images of the WT, TRV2: 00, (empty vector), TRV2: GhACX3 (silenced plants), and **(iv)** root morphological difference between WT, TRV2:00, and GhACX3 plants before drought and salt stress treatment. **(B)** Determination of **(i–iii)** plant height and root length, shoot fresh weight and root fresh weight of untreated, drought, and salt-treated plants. **(C)** Quantitative determination of **(i–iii)** excised leaf water loss, ion leakage, and relative leaf water content in untreated, drought, and salt-treated plants. Each experiment was repeated three times, bar indicates standard error (SE). Different letters indicate significant differences between wild-type and VIGS plants (ANOVA; *p* < *0.05*). Untreated indicates normal conditions.

We treated silenced *GhACX3* and control WT plants with drought and salt stress. Before the stress treatment, there was no significant differences observed on the various phenotypic variances measured but 24 h after drought and salt stress exposure, the plants exhibited a significant difference on the phenotypic variance (Figures 7Ai–iii). The leaves of silenced *GhACX3* had the wilted phenotype compared to the wild-type (WT). This showed that the knockdown of *GhACX3* significantly reduced the ability of the plants to tolerate the effects of drought and salt stresses.

**FIGURE 8:**
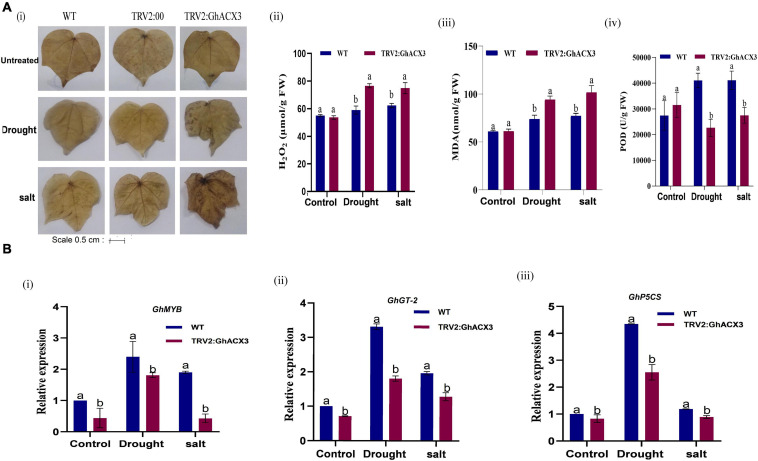
Biochemical analysis and RT-qPCR analysis of abiotic stress-responsive genes in Arabidopsis. **(A) (i)** Representative image of DAB staining assay of WT, TRV2: 00, and TRV2: GhACX3 in untreated, drought, and salt stress conditions, and quantitative determination of **(ii)** H_2_O_2_, **(iii)** MDA, and **(iv)** POD concentration in WT and silenced VIGS plants. **(B) (i–iii)** Relative expression of stress-responsive genes (GhMYB, GhTH, and GhP5CS) in silenced plants. Each experiment was repeated three times, and the bar indicates a standard error (SE). Different letters indicate significant differences between wild-type and VIGS plants (ANOVA; *p* < *0.05*). Untreated indicates normal conditions.

The fresh root (FR), shoot weights (SW), root length (RL), and plant height (PH) of silenced plants and the WT plants were evaluated. The root length of the wild-type exhibited higher growth or root extension compared to the VIGS plants (Figure 7Aiv), the higher root growth in wild-type was further evidenced by higher root biomass, no significant differences were observed among the untreated control for both VIGS and WT plants, however, under drought and salt stress conditions, the VIGS plants’ root biomass was significantly reduced compared to the WT or the positively controlled plants (TRV: 2) (Figure 7B). Moreover, similar trends were observed on other morphological parameters such the PH, and shoot fresh weight (Figure 7B). The results obtained were in agreement with previous findings in which knockdown of *LEA2* genes led to a reduction in both morphological and physiological traits under abiotic stress conditions (Magwanga et al., 2018b). We further evaluated various physiological parameters such as the excised leaf water loss (ELWL), relative leaf water content (RLWC), and cell membrane stability (CMS) in VIGS plants and WT. ELWL and ion leakage were higher in silenced plants compared to WT, while RLWC was lower in silenced plants relative to WT (Figures 7Ci–iii). We went further to determine the effect of Gh*_DO1GO186* knockdown by quantifying the oxidant and antioxidant activities. Under the simulated stress conditions, the oxidant levels were high, and the antioxidant levels were low in *GhACX3* plants compared to the WT. The silenced plants’ ability to tolerate the effects of drought and salinity stress was significantly reduced as evidenced by the DAB staining (Figure 8Ai); the VIGS plants had a high accumulation of hydrogen peroxide compared to the wild-type (Figure 8Aii). The MDA concentration level also increased more in silenced plants than in wild-type plants (Figure 8Aiii), whereas the antioxidant levels were significantly reduced in silenced plants (Figure 8Aiv). Previous studies have indicated that stress-responsive gene expression directly correlates to response to salt and drought stress situations. High expression of stress-responsive genes is a clear indication that the genes have a positive role in enhancing tolerance level to various abiotic stress factors (Butt et al., 2017; Magwanga et al., 2019a). In this study, the expression levels of known stress-responsive genes were determined; *GhMYB*, *GhG-T2*, and *GhP5CS*, previously found to have a higher functional role in enhancing plants response to various abiotic stress factors (Magwanga et al., 2019b), were evaluated. Their expression was significantly suppressed in the *GhACX3* knocked-down plants under drought and salt stress compared to the wild-type (Figure 8Bi–iii). These findings further confirm that knockdown of the *GhACX3* gene in plants compromised their ability to tolerate the effects of drought and salt stresses.

### Overexpression of GhACX3 Enhanced Tolerance to Drought and Salt Stress

**FIGURE 9:**
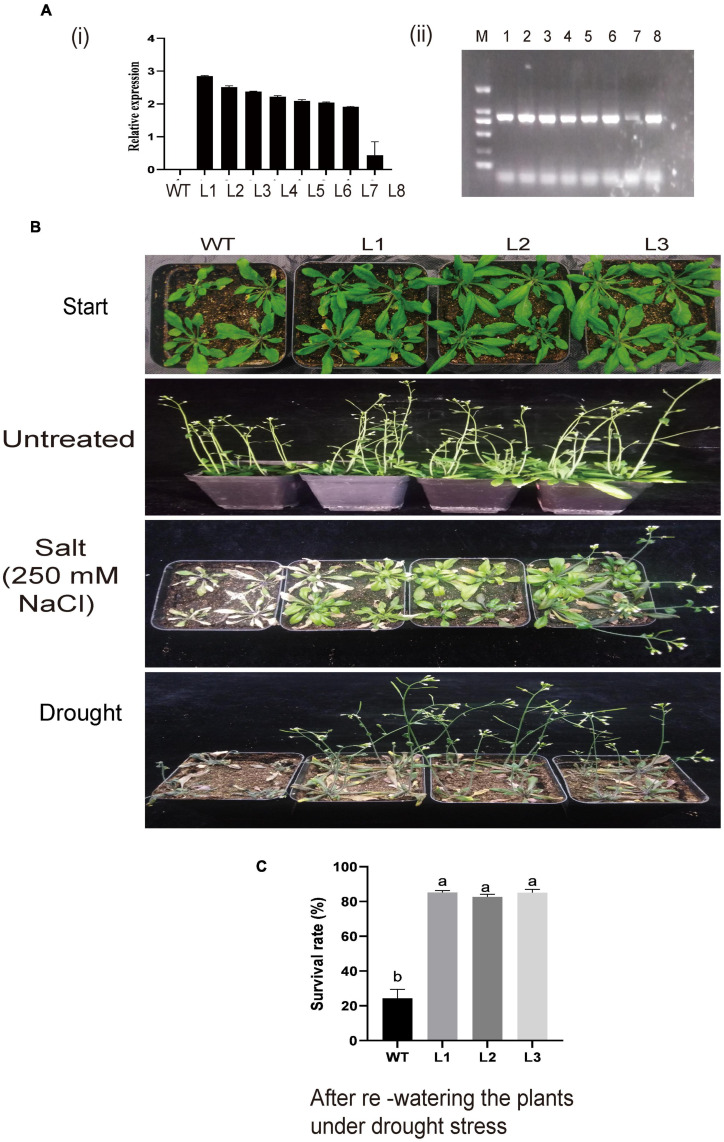
**(A)** Relative expression of overexpressed lines and phenotypic observation of the overexpressed plants. **(A) (i)** Relative expression of overexpression lines as determined by RT-qPCR. **(ii)** Semi-PCR analysis of the overexpressed lines. **(B)** Representative image of WT and L1, L2, and L3 overexpressed lines before treatment and under drought and salt treatment. **(C)** Comparison in survival rate between WT and overexpressed lines under drought stress after re-watering the plants.

Silenced *GhACX3* exhibited high susceptibility to these stresses; therefore, we went further to overexpress the gene in the model plant; Arabidopsis t was used to evaluate the plant’s stress response under these stresses. *GhACX3* was overexpressed in *A. thaliana* plants through the floral dip method. We selected the positively transformed plants from 50% MS with kanamycin. At T2, we selected the transformed lines through semi-quantitative RT-qPCR, and we choose three lines (L1, L2, and L3) for T3 generation (Figures 9Ai,ii). Phenotypically, all the plants showed no significant differences, but when the T3 lines were subjected to drought and salt treatment. The WT dried up under drought and salt stress, while the overexpressed lines were resilient and showed a higher level of survival (Figure 9B). The survival level of the plants after drought stress exposure through re-watering, revealed that survival rate of the overexpressed lines, L1, L2, and L3 were 9-fold higher compared to the wild-type (Figure 9C). The results obtained were in agreement with previous findings in which the overexpression of a novel G-protein-coupled receptors (GPCR) gene enhanced the ability of the model plant to tolerate the effects of salt stress (Lu et al., 2018).

**FIGURE 10:**
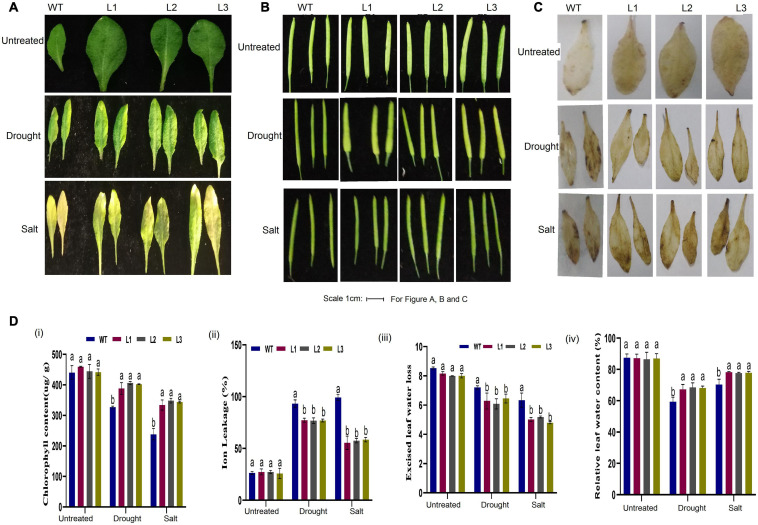
Phenotypic observation of leaf and seed pods and physiological analysis. **(A)** Representative leaf image of WT and overexpressed lines before and after drought and salt treatment. **(B)** Representative image of the seed pod of WT and overexpressed lines before and after drought and salt treatment. **(C)** DAB staining assay of WT and GhACX3-overexpressed lines in untreated, drought, and salt stress plants. **(D)** Quantitative determination of **(i)** chlorophyll content, **(ii)** ion leakage, **(iii)** excised leaf water loss, and **(iv)** relative leaf water content. Different letters indicate significant differences between wild-type and VIGS plants (ANOVA; *p* < *0.05*). Untreated indicates normal conditions.

**FIGURE 11:**
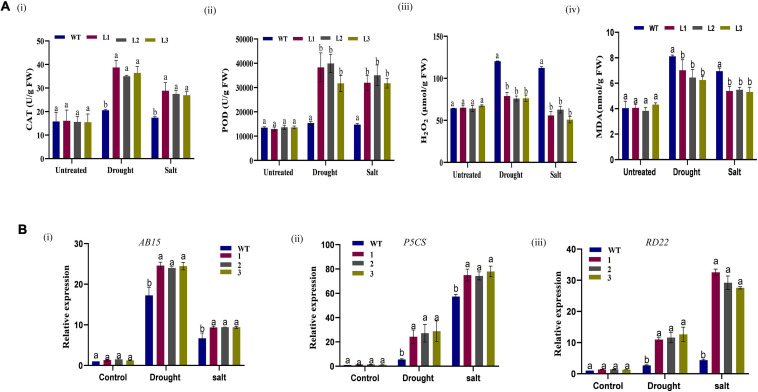
Biochemical analysis and qRT-PCR analysis of abiotic stress responsive genes in Arabidopsis plants. **(A) (i–iv)** Quantitative determination of CAT, POD, H_2_O_2_, and MDA in WT and overexpressed lines before and after stress treatment. **(B)** Relative expression of stress-responsive genes in WT and overexpressed GhACX3 Arabidopsis plants. **(i)** Relative expression of AB15, **(ii)** relative expression of P5CS, and **(iii)** relative expression of RD22. Error bars represent SD for three biological replicates. Different letters above the columns indicate significant differences at *P* < *0.05*. Untreated indicates normal conditions.

Moreover, the size and shape of the leaf and seedpod were evaluated; there was a significant difference between overexpressed and WT plants, an attribute which showed enhanced stress-tolerance (Figures 10A–C). To ascertain this phenotypic variance, we assessed the RLWC, ELWL, chlorophyll content, and ion leakage among the overexpressed lines L1, L2, and L3 compared to the wild-type. The ion leakage and ELWL levels were high in WT relative to overexpressed lines, while the RLWC and chlorophyll content was high in overexpressed *GhACX3* relative to WT (Figures 10Di–iv). We further evaluated the level of oxidant and antioxidant enzymes in overexpressed lines and the wild-type. The overexpressed lines registered significantly higher levels of antioxidant enzymes, compared to the wild-type (Figures 11Ai,ii), moreover, the oxidant levels were reduced under drought and salt stress conditions as evidenced by reduced malondialdehyde (MDA) and hydrogen peroxide (H_2_O_2_) concentrations in the overexpressed lines compared to control WT (Figures 11Aiii,iv). The results obtained were in agreement with previous studies which showed that the overexpressed lines were significantly well-adapted to the various stress factors, and were able to mobilize more of the antioxidant enzymes when exposed to various stress conditions (Kirungu et al., 2019; Magwanga et al., 2019a,c). Furthermore, the stress-responsive gene expression directly correlated to the plant’s drought and salt stress resilience (Finkelstein and Lynch, 2000; Silva-Ortega et al., 2008; Wang K. et al., 2012). In this study, we conducted RT-qPCR to profile the stress-responsive gene’s transcript level on overexpressed plants. *AtAB15*, *AtRD22*, and *AtP5CS*, these genes were significantly suppressed in WT compared to *GhACX3*-overexpressed Arabidopsis plants, were highly expressed (Figures 11Bi–iii).

### The Root Stress Tolerance Assay

**FIGURE 12:**
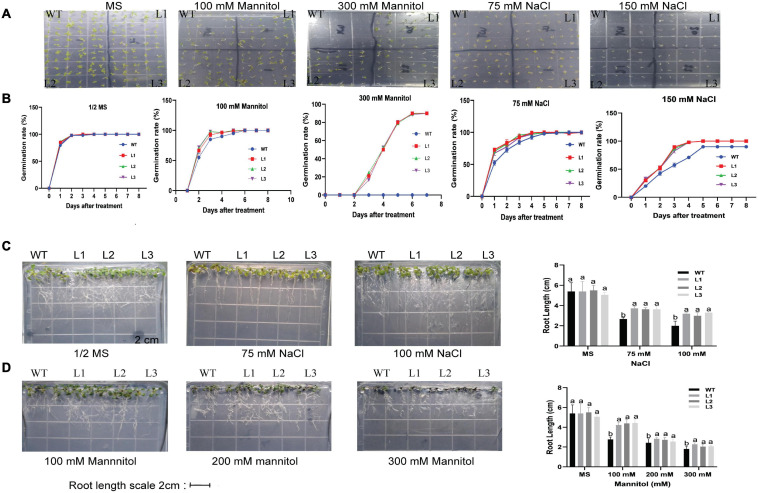
Root germination and elongation assay in Arabidopsis plants. **(A)** Analysis of germination in GhACX3-overexpressing lines and wild-type under stress conditions. **(B)** Phenotypic comparison of seedlings grown on 50% MS medium or MS with 100 mM of mannitol, 300 mM of mannitol, 75 mM of NaCl, and 150 mM of NaCl after 8 days. **(C,D)** The germination rate of the seedlings grown on MS medium and different concentrations of mannitol and NaCl. Analysis of root elongation in GhACX3-overexpressing lines and wild-type under MS added NaCl and mannitol and statistical analysis of the root lengths. Different letters above the columns indicate significant differences at *P* < *0.05*. MS represent normal conditions.

The seeds of overexpressed *GhACX3* and WT Arabidopsis were grown on MS medium with different NaCl and mannitol concentrations. Overexpression of this gene in Arabidopsis enhanced the seed resilience to salt and drought stress under different concentration levels. Even at higher stress levels, most overexpressed seeds were able to germinate, while most of the control seeds could not initiate root germination (Figures 12A,B). To determine the effect of stress on root development or elongation, we grew the seeds in a suitable growth medium for 6 days then transferred them to different concentrations of drought and salt stress to determine their tolerance levels. Under normal conditions, there was no significant difference in root development between overexpressed lines and WT. Nevertheless, after initiating stress, there was a clear significant difference in root development and elongation. The stress condition did not significantly hinder root development in overexpressed plants compared to WT (Figures 12C,D).

## Discussion

Drought and salt stress affect cotton plant optimal growth and development, ultimately resulting in low yields (Zulfqar et al., 2016; Odongo et al., 2020). An elaborated integrated molecular, cellular, and physiological response by the plant to abiotic stress ensures its survival and acclimation. Stress-responsive genes are involved in these critical mechanisms. Acyl-coenzyme oxidases 3, peroxisomal activities, and genes belonging to the *ACX* gene family, for a long time, have been thought to be primarily involved in the initial step of fatty acid β-oxidation. However, recent studies have proven that their functions have evolved and fatty acid β-oxidation occurs during various plant processes. This process occurs throughout the plant’s life cycle, and acyl-coenzyme A oxidase activities are upregulated in response to internal and external changes (Goepfert and Poirier, 2007). β-oxidation occurs in the process of IBA conversion to IAA (Ludwig-Müller, 2000), and in the production of jasmonic acid (Schilmiller et al., 2007) and hydrogen peroxide (Mano and Nishimura, 2005). Acetyl-CoA produced in β-oxidation is essential in histone acetylation (Wang et al., 2019) and also drives the cycle of tricarboxylic acid (TCA) for the aerobic production of ATP (Minard and McAlister-Henn, 1999; de la Peña Mattozzi et al., 2010); all these processes are in responses to abiotic stresses.

The advent and availability of the published genome sequence of cotton is undeniably a vital genomic resource in studying the gene’s evolutionary relationships. In this study, using the available published genome sequence, we identified several protein-encoding ACX3-conserved domain PF01756s. We identified 20, 11, and 9 proteins in *G. hirsutum* (AD) _1_, *G. arboreum* (A_2_), and *G. raimondii* (D_5_), respectively. Physiochemical properties identified in the cotton ACX3 included: CDS GC at an average 44.5% and negative grand average of hydropath (Gravy), among others. Negative Gravy is known as hydrophilicity. Hydrophilicity is an important feature associated with improving the protein membrane’s stability and osmotic modification during stress. This feature is also found in other stress-related genes like dehydrin in cotton (Kirungu et al., 2020).

We constructed a phylogenetic tree to study the evolutionary relationship of tetraploid cotton, diploid cotton, and other plant species. These genes evolved into three clades. The tetraploid cotton evolved closely together, with little variation as we observed. Notably, even their numbers were ranging between 18 and 22 proteins. The results indicate a close evolutionary relationship of genes that belong to the AD genome (tetraploids) relative to the A and D genome (diploids). The primary sources of gene-family enlargement in the cotton species are tandem and segmental replication, which occurs during polyploidization. Two or more genes, one after the other, on the same chromosome confirm tandem duplication events, while gene duplication is characterized as a segmental duplication event on multiple chromosomes. Duplication analysis of *ACX3* genes in cotton shows that these genes underwent segmental duplication. During evolution, duplicated genes undergo selection. The Ka/Ks ratio denotes the orientation and magnitude of the natural selection of protein-coding genes. A ratio greater than 1 means positive or Darwinian selection; less than 1 means selection purification or stabilization (acting against change); and a ratio of exactly 1 means selection neutral (i.e., no selection) (Kondrashov et al., 2002). In this study, we determined that this gene family undergoes purifying selection. Purifying selection is important during the evolution or duplication of genes as it ensures that genes that have an important role in the plant are maintained. These gene functions are stable throughout evolution (Article, 2018).

The intron-exon arrangement of genes in each genome was similar, and all had many introns. Introns form part of genome organization and function. The introns can house the regulatory elements and participate in initiating transcription and enhancing splicing (Rose et al., 2008). These are vital aspects that contribute to improved tolerance and plant adaptability to abiotic stress. *Cis-*regulatory elements identified were W-Box (WRKY-binding site), ABRE, CAAT–Box, TATA-box, MYB, MBS, TGA, LTR, TGACG, CGTCA-motif, G-Box, and Box-4 among others. All these are the binding sites for abiotic stress transcription factors, indicating that this gene is essential.

Gene Ontology analysis identified several GO terms associated with the gene family. The cellular component that denotes where all these functions occur was GO: 0005777; peroxisome. We went further and conducted bioinformatics prediction and found that in cotton, ACX3 is localized in the peroxisome, nucleus, mitochondrion, chloroplast, and endoplasmic reticulum. Peroxisomes are very dynamic and are involved in the cellular processes that include growth and development to signaling stress response (Sandalio, 2015). Peroxisomal activities involve interaction with other plant organelles in response to specific internal and external changes. Thus, peroxisomes are polyvalent organelles, and their functions differ significantly depending on cytoplasmic changes. For instance, fatty acid is transported to the peroxisomal for β-oxidation to occur and the products are precursors of different cellular processes. Their availability depends on cytoplasmic changes (Grevengoed et al., 2014).

The differential gene expression in different organs and tissue under stress can indicate the gene family’s function. In this research work, we studied the expression pattern of *GhACX3* genes under stress. We found that most of the genes were highly upregulated in the roots and differentially expressed in the leaf and stem. These outcomes agree with a previous study in Arabidopsis that found *AtACX3* highly expressed in the root (Eastmond et al., 2000). Roots have the ability to sense the change in the soil’s osmotic potential, thereby initiating an appropriate response to drought and high salinity (Perlikowski et al., 2020). The process of sensing and signal transduction plays a critical role in initiating specific plant responses to stress. The *ACX3* gene is directly involved in the conversion of IBA to IAA; these phytohormones are involved in root elongation. This was demonstrated by studying the effect of acx3 Arabidopsis mutant plants on IBA and was found to interfere with its responsiveness (Strader et al., 2010). Once *ACX3* converts IBA to IAA, IAA stimulates mechanisms that stimulate some phytohormones-related genes like auxin, abscisic acid, jasmonic acid, and halts the effect of drought and salt stress (Schlicht et al., 2013; Pandey, 2017).

The VIGS assay and overexpression assay for loss and gain of function mutations has been used to study the function of genes in plants. We employed both assays to study the role of *GhACX3* in drought and salt stress conditions. Plant stress inhibits plant optimal growth and development. These result in changes in transpiration rate, development, and composition of photosynthetic apparatus, increased ROS production, and biochemical composition changes. In this study, loss of function caused the plants to be more vulnerable to drought and salt stress. This was well depicted with the phenotypic difference between control and silenced plants, lower relative leaf water content (RLWC), and shoot fresh weight (SFW) compared to control WT. This indicated that the silenced plants were experiencing a high transpiration rate. Lipid peroxidation and high hydrogen peroxide are markers for oxidative stress in plants. Increased oxidation, which is often evaluated through MDA, H_2_O_2_, and the DAB assay, correlates to increased susceptibility of plants to drought and salt stress. This is because the high production of oxidants in plants indicates the plant’s compromised or weak mechanisms of producing antioxidants. Antioxidants play an essential role in maintaining homeostasis in plant cellular organs, like the chloroplast, peroxisome, and mitochondria that produce oxidants, thereby offering the plant defense against oxidative stress (Maryam et al., 2012). Low concentrations of antioxidants (CAT and POD) and high concentrations of oxidants (MDA and H_2_O_2_) correlate to compromised plant mechanisms to maintain a homeostatic environment in its cell compartments. Therefore, the plant produces a high amount of ROS, which damages the plant’s photosynthetic apparatus. As a result, the cell membrane is damaged; as demonstrated from this study, silenced plants had a high ion leakage and lower chlorophyll content relative to control WT.

In contrast, *GhACX3*-overexpressed Arabidopsis plants had enhanced tolerance to drought and salt stress. The plants had a high survival rate after re-watering the plants, which were under water withdrawal. Physiological parameters evaluated on overexpressed plants under drought and salt stress demonstrated high water retaining capability due to a lower transpiration rate. Evident from this experiment, RLWC was high and ELWL was lower relative to control WT. The ion leakage was also lower relative to control WT. Further we analyzed oxidants (MDA and H_2_O_2_) and antioxidants (CAT and POD) levels. Overexpressed plants visibly demonstrated lower oxidants and a high concentration of antioxidants relative to control WT. When we planted the Arabidopsis seeds of WT and *GhACX3* lines under drought and salt stress, overexpressed plants exhibited high tolerance by having higher root germination and elongation rate relative to WT. Evidently, from this study, *GhACX3-*overexpressed plants had improved drought and salt stress tolerance.

Under drought and salt stress conditions, plants have evolved and initiate complex mechanisms to enhance survival strategies. This mechanism also involves high expression of transcription factors and stress-responsive genes. *GhMYB*, *GhT2*, and *GP5CS* are stress-related genes studied in cotton plants. From the study of these genes, the researchers concluded that they significantly participated in cotton plant tolerance and acclimation to abiotic stress (He et al., 2016; Magwanga et al., 2019a) and (Dobrá et al., 2011). In this study, we examined the expression of these genes in silenced *GhACX3* plants, and the expression was relatively regulated compared to WT. These findings show that the silencing of *GhACX3* in the cotton plant comprised the plant’s ability to initiate a stress response.

Similarly, we examined the expression of the *AtAB15*, *AtP5C5*, and *AtRD22* stress-related genes in overexpressed plants. The expression of *AtAB15*, *AtP5C5*, and *AtRD22* in *GhACX3*-overexpressed plants was high from our research. The results demonstrated that the overexpression of *GhACX3* in Arabidopsis enhanced the regulation of other genes responsive to drought and salt stress. Previous studies on stress-related genes showed that these genes’ overexpression enhanced drought and salt tolerance in cotton and other plants (Finkelstein and Lynch, 2000; Silva-Ortega et al., 2008; Wang H. et al., 2012).

## Conclusion

These research findings have shown that plant ACX3 protein plays a role in enhancing drought and salt tolerance. Overexpression of this gene enhanced tolerance under stress, which was exhibited by the germination assay. The overexpressed seed growth rate was faster relative to control under stress conditions. The survival rate was also higher in overexpressed plants relative to control plants under drought stress. In contrast, silencing the *GhACX3* gene in cotton plants resulted in plants showing a stress susceptibility phenotype. These findings provide fundamental molecular knowledge on the role of ACX3 in drought and salt tolerance and can be exploited further for genetic improvement and to breed drought- and salt stress-resilient cotton varieties.

## Data Availability Statement

The original contributions presented in the study are included in the article/Supplementary Material, further inquiries can be directed to the corresponding author/s.

## Author Contributions

RM and FL: conceptualization and validation. MS: methodology, formal analysis, and investigation. MS, RM, YX, and TM: software. XC, YH, and YW: resources. RM: data curation. MS and SA: writing—original draft preparation. RM, JK, SA, and MS: writing—review and editing. RP: visualization. FL: supervision and project administration. YW, KW, and FL: funding acquisition. All authors have read and agreed to the published version of the manuscript.

## Conflict of Interest

The authors declare that the research was conducted in the absence of any commercial or financial relationships that could be construed as a potential conflict of interest.

## Publisher’s Note

All claims expressed in this article are solely those of the authors and do not necessarily represent those of their affiliated organizations, or those of the publisher, the editors and the reviewers. Any product that may be evaluated in this article, or claim that may be made by its manufacturer, is not guaranteed or endorsed by the publisher.

## Footnotes

1
http://www.web.xpasy.org/compute_pi/

2
www.cottonfgd.org

3
http://gsds.cbi.pku.edu.cn

4
http://meme-suite.org

## Supplementary Material

Supplementary Table 1 Physiochemical properties of the proteins encoded by the *GhACX3* genes.

Supplementary Table 2 Selective pressure of homologous genes among *G. arboreum, G. raimondii* and *G. hirsutum.*

Supplementary Table 3 List of all the primers used in this study.
